# Relative Entropy-Based Reliability Assessment of Hybrid Telecommunication Skeletal Towers

**DOI:** 10.3390/e28020137

**Published:** 2026-01-25

**Authors:** Marcin Kamiński, Rafał Bredow

**Affiliations:** Department of Structural Mechanics, Faculty of Civil Engineering, Architecture & Environmental Engineering, Lodz University of Technology, 93-590 Lodz, Poland

**Keywords:** hybrid telecommunication towers, reliability assessment, metal structures, Stochastic Finite Element Method, probabilistic entropy

## Abstract

The main aim of this paper is the uncertainty quantification and reliability assessment of the hybrid skeletal telecommunication tower subjected to dynamic wind pressure. The structural response of this aluminum–steel construction is contrasted with the original steel tower solution widely available in engineering practice in the numerical environment of the system ABAQUS 2024. Some design parameters of both towers are considered uncertain and distributed according to the Gaussian probability distribution so that the resulting reliability indices in the Ultimate Limit State (ULS), as well as the Serviceability Limit State (SLS), are determined. These indices are calculated using the First Order Reliability Method (FORM), and also from the probabilistic entropy scheme due to the Bhattacharyya theory. The first two probabilistic characteristics necessary for the reliability assessment result from the Stochastic Finite Element Method implemented according to the generalized iterative stochastic perturbation technique. All probabilistic calculus is programmed in the symbolic algebra of the system MAPLE 2015. As it is documented in this study, a choice of the hybrid tower enables for some mass savings under preservation of the same reliability level.

## 1. Introduction

Structural safety is a matter of primary importance, especially in the context of various optimization issues. The influence of multiple random design parameters, their statistical dispersions, and cross-correlations on structural safety is the main concern of the reliability theorem. Multiple literature positions are dedicated to the reliability assessment and its fundamentals; see the works written by Melchers [1], Ditlevsen & Madsen [2], and Nowak & Collins [3]. The first attempt to express structural reliability in the form of an index was submitted by Cornell [4]. This idea has been further expanded by Hasofer & Lind [5] and Rackwitz & Fiessler [6], where the reliability index has been described by the shortest distance to a point on the failure surface from the origin of the standard normal space (U-space). The so-called First-Order Reliability Method (FORM) assumes that the limit state function in the U-space is linearized by the first-order Taylor expansion at the design point, whereas the second-order reliability method (SORM) approximates the limit state function by the second-order Taylor expansion at the design point. This contributes to the fundamental distinction of these two groups of methods, where in FORM the limit state surface is expressed by a linearized plane and for SORM by a paraboloid. There are also approaches based on the method of moments [7,8,9], and the structural reliability assessment is most commonly based upon these methods. Their accuracy and computational efficiency are broadly discussed in numerous works [10,11,12,13]. Currently, valid codes for structural design state requirements concerning structural safety, serviceability, and durability also give guidelines for structural reliability aspects. Structural reliability aspects can be differentiated by the Consequences Classes (CC), and they can be associated with the Reliability Classes (RC), which are in turn expressed in the form of the specific values of the reliability index. Appendix C of Eurocode 0 indicates some limit values of reliability index for the Ultimate Limit State, Serviceability Limit State, or even Fatigue Limit State for a certain reference period, whereas Annex B of Eurocode 0 contains some minimum numerical values of this index depending on both RC and reference period. Appendix A of Eurocode 0 additionally states a series of guidelines for the assessment of several SLS related to vibration, but these states are related to pedestrian comfort on footbridges or some verification procedure on the deformation and vibration of railway and road bridges. It should be underlined that there are no explicit comments specifying how to approach a problem of structural dynamic excitation at ULS and SLS in a more general concern; for example, in residential buildings, warehouses, and other industrial objects. This problem gains specific importance for the skeletal structures (towers, masts) when time-varying processes are considered. Dynamic analysis of structures focuses on time-variant response concerning subjected time-varying loads/excitations, specifically when the acceleration of the system cannot be neglected [14]. A set of equations of motion obtained due to the implementation of Finite Element Method (FEM) at each time step of dynamic analysis can be computed with implicit or explicit methods. Implicit methods generally bind nodal displacement and velocity vectors of a subsequent time step with a current time step, and they exhibit unconditional stability. Explicit dynamic analysis, on the other hand, computes vectors of nodal displacements and velocities with known values of nodal displacement, velocity, and acceleration vectors from the previous time step only. A choice between explicit and implicit methods strongly depends on the time variability of the dynamic load. Traditional structural engineering problems may be frequently solved with the use of implicit methods, whereas explicit methods are preferred when a dynamic phenomenon related to impact loads is suggested. For the explicit method, it is crucial to set properly small time steps of computations, partially due to the conditional stability of the method. It is recommended to make an approximation of the admissible maximum size of the time step based on the speed of the longitudinal sound wave that propagates in the investigated material. When such data are not accessible, some further and more general estimations can be made based on material density and elastic moduli. Computational time steps can be relatively large for the implicit method applications, but convergence is expected at all the time steps. The global equilibrium state of the system is achieved at each time step, which is obtained through the iteration process, then the system computes local finite element variables like stresses. On the contrary, for explicit analysis, no global equilibrium state is verified (no convergence criteria to check), and the system pursues straightforward estimation of the finite element variables. There are many implicit methods proposed in the literature such as Newmark [15], Euler, Crank-Nicolson [16], Runge-Kutta, Adams, Newmark-Bossak [17], Wilson, Park-Housner [18], and the space-time element method [19,20,21]. Within the scope of FEM, very commonly implicit methods consist of the Newmark algorithm [15], but also within the further development of numerical methods related to dynamic problems, different approaches have been proposed. One of them is a Hilber-Hughes-Taylor algorithm [22], which is a modified Newmark method, where numerical damping is involved, which additionally improves the accuracy of the method and lowers the accuracy loss with some prevention of the numerical ringing phenomenon.

Recent accessibility to the FEM software and its current ongoing development, along with significant growth of computing power, led to a substantial improvement of possibilities to conduct numerous optimization processes, but also stochastic analysis. The Stochastic Finite Element Method (SFEM) gained popularity as its contribution to uncertainty quantification and reliability analysis achieved widely spread recognition. Engineering experience and intuition, as well as accumulated data, show that uncertainties have their source not only in the loads and their nature but also in the geometrical features and mechanical properties of the materials and the overall structure itself. Nowadays, civil engineering structures are designed with the use of some minimum, maximum, or mean representative design values.

A remarkable advance in the numerical simulations, together with the computational capability of personal computers, allowed for the analysis of significantly more complex structures not only in deterministic, but also in the probabilistic approach. Modern probabilistic computational mechanics include a variety of Monte-Carlo simulations, Karhunen-Loéve [23] and polynomial chaos expansions, various order perturbation methods, interval sampling, and fuzzy sets theory, as well as even some non-traditional methods and models [24]; some recent papers additionally introduce Probability Transformation Methods (PTM) [25]. These methods are of specific importance in geotechnical engineering, where statistical dispersions quite naturally seem to be remarkably larger than in structural studies [26], especially in the case of steel structures. Excellent mechanical properties of metal structures allow the design of very slender structures, which very commonly suffer from global and local stability loss [27,28]. Due to the significant slenderness of metallic structures, large displacements are expected; thus, study on their behavior should concern the problems of geometrically nonlinear response, which brings additional computational effort [29]. Metal structures very commonly are shaped as trusses, which are applied in the civil engineering field, and they commonly are applicable in the bridge spans, roof truss-purlins, or even telecommunication towers; truss structures are widely studied in terms of their non-linear response and reliability [30,31,32]. It is obvious that telecommunication towers are widely recognized for their strategic meaning worldwide. Their reliability and damage prediction are objects of study [33,34]. Also, their dynamic behavior, mostly induced due to wind action, is a matter of primary significance to their reliability and overall durability [35,36]. Additionally, traditional structural steel grades are very sensitive towards their degradation due to corrosion processes. Skeletal towers and other slender tall structures are very expensive in their lifespan due to the repetitive conservation regarding anticorrosive coating maintenance. For this reason, it seems reasonable to construct their highest segments from aluminum. Hence, it is not corrosive, and it reduces the maintenance costs even though such solution also implicates additional initial costs during the construction process since aluminum is very often more expensive material on the worldwide market. Among the other reasons, this is the main motivation to investigate further steel towers and aluminum–steel towers (hybrid towers) as two examples of such strategic objects.

A hybrid steel–aluminum telecommunications tower subjected to a dynamic wind pressure distribution having uncertain characteristic velocity is the subject of this study [37]. It is designed with lower segments manufactured with stainless steel and upper ones, with the use of some aluminum alloys. Such a structural solution allows to remarkably reduce the overall weight of the tower while comparing with an analogous, original steel tower of the same height and signal transmission functionality. The basic Finite Element Method (FEM) model of this tower has been completed in the commercial system ABAQUS^®^ 2024 using displacement-based formulation, its 3D beam and truss linear elements, and also elastic supports. It reflects calibration of the numerical model to the experimental push-over test reported in [38]. The dynamic response of the tower in the context of displacement and stress histories has been determined numerically using the Hilber-Hughes-Taylor (HHT) and contrasted with the results obtained from the classical Newmark scheme. Probabilistic analysis has been completed using the iterative generalized stochastic higher-order perturbation technique, where dynamic structural polynomial response functions have been found by an application of the Weighted Least Squares Method (WLSM) [39] and a special series of the FEM experiments. The LSM approximation procedures, as well as all non-deterministic procedures, have been implemented in the environment of computer algebra system MAPLE 2015^®^. The expected values, coefficients of variation, and skewness, as well as kurtosis of extreme tower displacements and reduced stresses, were used in the final reliability assessment. The following random input parameters have been considered in this study: antenna masses, soil compliance, environmental temperature, and structural round pipes thicknesses, as well as wind characteristic velocity. According to the fact that they have totally different physical nature, no cross-covariances have been considered. Gaussian probability distributions are adopted for all these parameters, because for the given expectation and standard deviation, it causes the largest entropy (disorder) in the parameters subjected to randomization; this fact follows directly the Maximum Entropy Principle.

The basic engineering reliability analysis has been provided here with the use of the First Order Reliability Method (FORM) recommended by Eurocode 0, and this approach has been contrasted with the probability distance model provided by Bhattacharyya [40,41]. As it is known, the FORM apparatus is the most convenient for linear limit functions or the functions that can be linearized only, whereas general reliability assessment with possibly non-Gaussian responses, where the first two moments may be not representative, demanding a more general approach like the entropic one proposed here. The Bhattacharyya distance seems to be a more appropriate measure to study the differences in-between admissible and extreme probability distribution functions inherent in the Serviceability and Ultimate Limit States, especially when the structural response exhibits a non-Gaussian distribution. A literature reporting a usage of the relative entropies proposed by Kullback-Leibler [42] and Bhattacharyya [43,44] in traditional reliability assessment are rather scarce and still not discussed in the context of wider spectrum of realistic engineering structures.

Nevertheless, some analytical expression similar to the FORM analysis has been provided. A special upscaling procedure has been successfully proposed because such a probabilistic distance exhibits the same sensitivity concerning the input uncertainty level as the FORM index but shows different numerical values range. It has been demonstrated that the hybrid aluminum–steel towers are an interesting alternative to traditional steel structures, and also that the probability distance approach proposed may be used instead of the FORM approach in many engineering reliability studies. A key novelty in this study is dual reliability assessment of the hybrid steel–aluminum towers delivered using three essentially different probabilistic numerical techniques, where the reliability index is not calculated from the wind pressure static equivalent but directly, from the experimentally driven wind forces variations.

## 2. Numerical Model Equations

Let us consider the Reliability-Based Design Optimization (RBDO) problem [45] of the metal skeletal tower made of a composition of the beam (edge elements) and bar elements (stiffeners). It is subjected to various uncertainties in environmental actions (wind and temperature variations), as well as live loads (equipment mass) and material (i.e., Young modulus) or geometrical parameters. These uncertainties are represented by the vector $b_{r},\;r=1,\ldots ,R$, whose components are assumed as uncorrelated due to the lack of any experimental evidence. An optimization cost function is traditionally the dead load of the entire tower $w=w\left(b_{r}\right)$ and is subjected to a minimization procedure, where the following constraints apply:

(1) $$\beta_{SLS}-{\hat{\beta }}_{SLS}=\beta_{SLS}\left(b_{r}\right)-{\hat{\beta }}_{SLS}\geq 0,\;r=1,\ldots ,\;R,$$

(2) $$\beta_{ULS}-{\hat{\beta }}_{ULS}=\beta_{ULS}\left(b_{r}\right)-{\hat{\beta }}_{ULS}\geq 0,\;r=1,\ldots ,\;R.$$

Here $\beta_{SLS},\;\beta_{ULS}$ mean serviceability and ultimate limit states reliability indices, whereas ${\hat{\beta }}_{SLS},\;{\hat{\beta }}_{ULS}$ are their admissible counterparts. The indices $\beta_{SLS},\;\beta_{ULS}$ may be determined using various procedures, and the First Order Reliability Method [4] and relative entropy-based method have been contrasted in this work. The FORM approach is based on the following definitions:

(3) $$\beta_{SLS}\left(b_{r}\right)=\frac{E\left[q_{R}\right]-E\left[q_{E,\max}\left(b_{r}\right)\right]}{\sigma \left(q_{E,\max}\left(b_{r}\right)\right)},$$

(4) $$\beta_{ULS}\left(b_{r}\right)=\frac{E\left[f_{y}\right]-E\left[\Sigma_{E,\max}^{red}\left(b_{r}\right)\right]}{\sigma \left(\Sigma_{E,\max}^{red}\left(b_{r}\right)\right)},$$

where $q_{R}$ and $f_{y}$ denote admissible horizontal displacement of the tower’s top and yield strength of its most efforted cross-section; *R* denotes here the total number of various uncertainty sources. Further, the quantity $q_{E,\max}\left(b_{r}\right)$ stands for the extreme horizontal displacement of the tower’s top over the given time interval, and the extreme reduced stress $\Sigma_{E,\max}^{red}\left(b_{r}\right)$ in Equation (4) is determined using the Huber-Mises-Hencky strength hypothesis via the FEM procedure. Extreme displacement $q_{E,\max}\left(b_{r}\right)$ is determined from the following equation of motion [16]:

(5) $$\begin{array}{l} M{\ddot{q}}_{n+1}+(1+\alpha_{HHT})C{\dot{q}}_{n+1}-\alpha_{HHT}C{\dot{q}}_{n}+(1+\alpha_{HHT})Kq_{n+1}-\alpha_{HHT}Kq_{n}\\ \;=F(t_{n}+(1+\alpha_{HHT})\Delta t) \end{array}$$

It links displacements $q_{n+1}$, velocities ${\dot{q}}_{n+1}$, and accelerations vectors ${\ddot{q}}_{n+1}$ for $\left(n+1\right)\Delta t$ time instant; it includes the mass, damping, and stiffness matrices **M**, **C**, and **K**, the external loadings vector **F** inherent in the FEM, while $\alpha_{HHT}$ is the coefficient in the Hilber-Hughes-Taylor method enabling efficient solution of this equation. Moreover, the operators $E\left[.\right],\;\sigma \left(.\right)$ are the expectation and standard deviation of the given extreme structural responses, and they are determined starting from the following the WLSM based polynomial approximants:

(6) $$q_{E,\max}\left(b_{r}\right)=Q_{0}^{\left(r\right)}+Q_{1}^{\left(r\right)}b_{r}+Q_{2}^{\left(r\right)}b_{r}^{2}+\ldots +Q_{p}^{\left(r\right)}b_{r}^{p},$$

(7) $$\Sigma_{E,\max}^{red}\left(b_{r}\right)=S_{0}^{\left(r\right)}+S_{1}^{\left(r\right)}b_{r}+S_{2}^{\left(r\right)}b_{r}^{2}+\ldots +S_{p}^{\left(r\right)}b_{r}^{t}.$$

The coefficients $Q_{0}^{\left(r\right)},Q_{1}^{\left(r\right)},Q_{2}^{\left(r\right)},\ldots ,Q_{p}^{\left(r\right)}$ and $S_{0}^{\left(r\right)},S_{1}^{\left(r\right)},S_{2}^{\left(r\right)},\ldots ,S_{p}^{\left(r\right)}$ are found from the series of the FEM experiments with specifically varying input parameters, while *p* and *t* represent statistically optimized polynomial bases orders. Such an optimization procedure obeys the determination of the few polynomial bases having increasing orders starting from a linear one. Then, the optimal choice is equivalent to the specific order, which minimizes approximation RMS error and maximizes its correlation factor. Finally, the basic two probabilistic characteristics inherent in the Formulas (3) and (4) are determined using three alternative stochastic techniques. The first one, called the semi-analytical approach (SAM), is based on the analytical symbolic calculus, where

(8) $$E\left[q_{E,\max}\left(b_{r}\right)\right]=\int_{-\infty }^{+\infty }q_{E,\max}\left(b_{r}\right)\;p\left(b_{r}\right)(x)\;dx,$$

and also

(9) σqE,maxbr=∫−∞+∞qE,maxbr−EqE,maxbr2 pbr(x) dx12.

This method works efficiently if only these integrals exist and may be determined. Otherwise, the second approach is recalled, which unconditionally works, and this is the stochastic perturbation technique (SPT) [46], where the random structural output is expanded about its mean value, with the Taylor series having random coefficients as follows:

(10) $$q_{E,\max}\left(b_{r}\right)=q_{E,\max}^{0}\left(b_{r}^{0}\right)+\sum_{j=1}^{N}\sum_{r=1}^{R}\frac{\varepsilon^{j}}{j!}\frac{\partial^{j}q_{E,\max}\left(b_{r}^{0}\right)}{\partial b_{r}^{j}}{\left(b_{r}-b_{r}^{0}\right)}^{j};$$

*N* denotes the chosen order in the SPT expansion, *ε* is the perturbation parameter, and the upper index 0 stands for the mean values of the given random functions and parameters. Let us note that polynomial bases apply to $q_{E,\max}\left(b_{r}\right)$ guarantee unconditional convergence of this expansion, contrary to a divergence of the general Taylor series case. The last method, which is frequently recommended as the referential one, is the Monte-Carlo simulation (MCS) approach, where modern random number generators (like Mersenne twister, for instance [47]), together with accelerated statistical estimators, are employed. It yields for *M*, denoting the total number of random experiments

(11) $$E\left[q_{E,\max}\left(b_{r}\right)\right]=\frac{1}{M}\sum_{m=1}^{M}q_{E,\max}\left(b_{r}^{\left(m\right)}\right),$$

and

(12) σqE,maxbr=1M−1∑m=1MqE,maxbr(m)−EqE,maxbr212.

Determination of the first two probabilistic characteristics of the extreme reduced stress proceeds in a similar triple way. Finally, the FORM reliability indices are calculated using Equations (3) and (4) and contrasted with the Bhattacharyya relative entropy model. As it was mentioned, relative entropy quantifies a divergence of two probability distributions and, in this context, seems to be more justified than the FORM approach in reliability assessment. Therefore, one may rewrite

(13) $$\begin{array}{l} H_{SLS}\left(b_{r}\right)=\frac{1}{4}\frac{{\left(E\left[q_{R}\right]-E\left[q_{E,\max}\left(b_{r}\right)\right]\right)}^{2}}{{\left(\sigma \left(q_{E,\max}\left(b_{r}\right)\right)\right)}^{2}+{\left(\sigma \left(q_{R}\left(b_{r}\right)\right)\right)}^{2}}\\ \;\;\;\;\;\;\;\;\;\;\;\;\;\;\;\;\;\;\;\;\;\;\;\;\;\;\;\;\;\;\;\;\;\;\;\;\;\;\;\;+\frac{1}{2}\ln\left(\frac{{\left(\sigma \left(q_{E,\max}\left(b_{r}\right)\right)\right)}^{2}+{\left(\sigma \left(q_{R}\left(b_{r}\right)\right)\right)}^{2}}{2\sigma \left(q_{E,\max}\left(b_{r}\right)\right)\sigma \left(q_{R}\left(b_{r}\right)\right)}\right), \end{array}$$

(14) $$H_{ULS}\left(b_{r}\right)=\frac{1}{4}\frac{{\left(E\left[f_{y}\right]-E\left[\Sigma_{E,\max}^{red}\left(b_{r}\right)\right]\right)}^{2}}{{\left(\sigma \left(\Sigma_{E,\max}^{red}\left(b_{r}\right)\right)\right)}^{2}+{\left(\sigma \left(f_{y}\right)\right)}^{2}}+\frac{1}{2}\ln\left(\frac{{\left(\sigma \left(\Sigma_{E,\max}^{red}\left(b_{r}\right)\right)\right)}^{2}+{\left(\sigma \left(f_{y}\right)\right)}^{2}}{2\sigma \left(\Sigma_{E,\max}^{red}\left(b_{r}\right)\right)\sigma \left(f_{y}\right)}\right).$$

A specific rescaling procedure of these entropies to the real-valued interval of the FORM index is proposed here in a way verified in the literature before. It yields

(15) $$\beta_{SLS}^{H}\left(b_{r}\right)=2\sqrt{H_{SLS}\left(b_{r}\right)},$$

(16) $$\beta_{ULS}^{H}\left(b_{r}\right)=2\sqrt{H_{ULS}\left(b_{r}\right)}.$$

One concludes that if extreme or admissible values are deterministic, then the FORM and relative entropy indices are simply equal to each other.

## 3. Numerical Experiments

**Figure 1:**
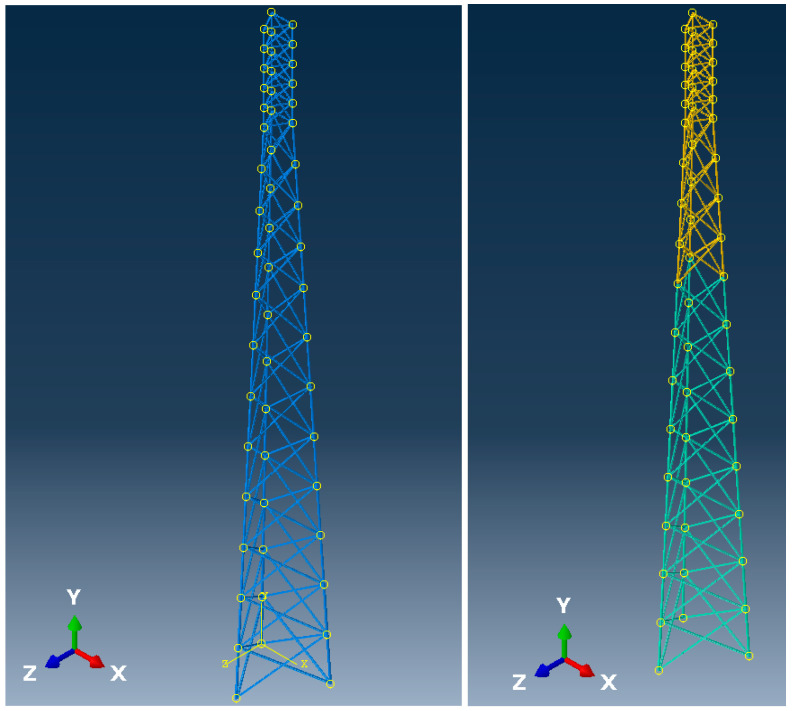
Visualization of the skeletal tower geometry ((**left graph**)—steel tower; (**right graph**)—hybrid tower, steel elements—green, aluminum elements—yellow).

The overall geometry of the investigated structure under consideration represents some skeletal towers commonly used in the telecommunication industry as base towers. Both numerical structures have been modeled as 3-legged lattice towers. The first structure has been modeled with the use of steel cross sections [38,48], while the second one has been modeled with two kinds of materials—the lower part (inclined segment) from steel cross sections identical to the first tower, and the upper part from aluminum cross-sections. Both investigated structures exhibit identical nodal geometry. The main legs have been modeled as circular hollow sections and bracing elements as the angle bars. The bottom part of the skeletal tower exhibits the inclination of main legs from the vertical orientation, and the upper part has parallel main legs. The height of the bottom part of the tower is equal to 34.0 m, whereas the upper part has a height equal to 6.0 m. The overall height of the investigated tower is then equal to 40.0 m. Geometrical specification and cross-section assignments to beam members are shown in Table 1 for the steel tower and in Table 2 for the hybrid (steel + aluminum) tower. They have been proposed and established to achieve about 90% effort of the key structural elements throughout the heights of both towers; visual representation of the geometry is shown in Figure 1.

**Figure 2:**
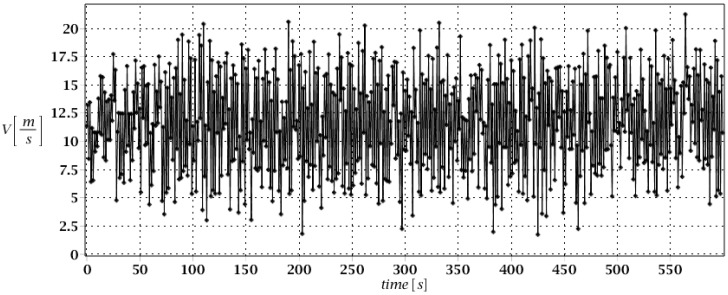
Wind load history in 10-min time interval.

**Figure 3:**
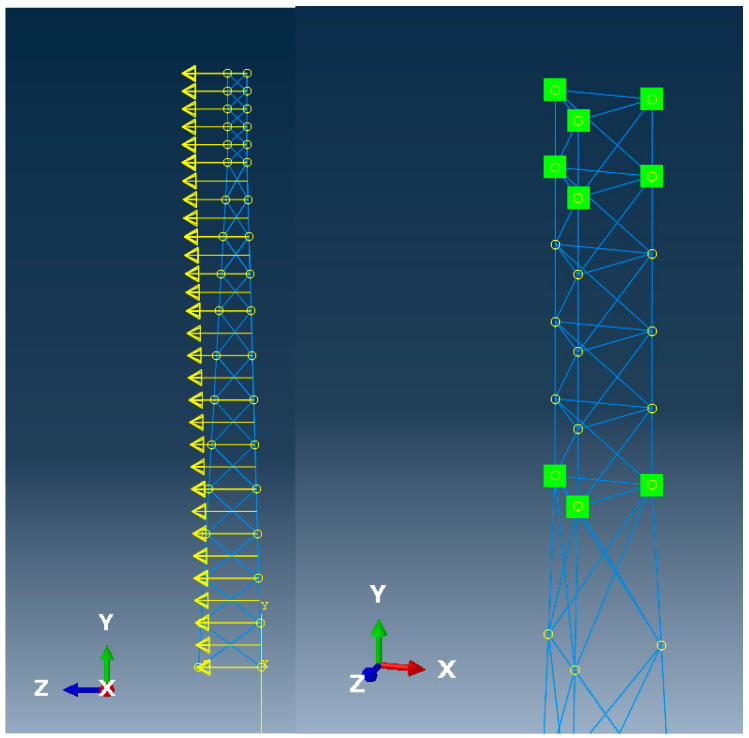
Wind load distribution (**left**, yellow arrows) and the nodes with concentrated masses (**right**, green squares).

Numerical models for both investigated types of skeletal towers have been created in the ABAQUS 2024 system. Main legs and bracing elements have been modeled as linear beam elements in space named B31 finite elements based on Timoshenko formulation [49] (shear-flexible beam elements). The model consists of 882 nodes and 996 finite elements, resulting in a total number of equations exceeding 5200. At the bottom of the skeletal tower, spring pin supports have been prescribed, involving some additional interrelation of actions between the foundation of the skeletal tower and subsoil. The system has been modelled as located on the Winkler type of the subsoil, which is driven by the designing practice, and also by some calibration versus the full-scale push-over test [38]. A deadload of the structure has been introduced into the numerical model, primarily with some additional concentrated mass points representing additional telecommunication equipment on the top segments of the tower. These equipment masses have been described by their weight and, also, moment of inertia to more accurately assess their influence on the vibrations of structure. Besides static loads described above, skeletal towers have been subjected to dynamic wind excitation in 10-min time intervals described by some wind spectrum shown in Figure 2. This wind action on the structure has been, firstly, assessed as quasi-static, following Eurocodes guidelines [50], especially for towers, masts, and chimneys [51]. The concentrated forces have been applied to the nodes with concentrated masses simulating the aerodynamic resistance of telecommunication equipment. Quasi-static wind pressure has been transformed to the dynamic action with the use of experimentally determined spectrum available in the literature [52]. The additional 10-min time interval is given in Figure 2, accordingly. Spatial distribution of the mean wind forces acting on the structure has been shown in Figure 3 (left graph), whereas the location of nodes where telecommunication equipment has been prescribed has been shown in right graph of Figure 3.

The maximum time step of solution finding has been set as 0.5 s, and the ABAQUS 2024 system automatically sets the current time step according to iteration steps, providing the desired accuracy of the method based upon the half-time increment residual when necessary [53]. Also, desired structural response values have been set to be recovered at strictly chosen time points of analysis every 0.5 s, meaning, finally, for each desired structural response type, there have been 1200 discrete values to be recovered. This aforementioned dynamic analysis has been carried out concerning six independent Gaussian design uncertainty sources, and for each of them, 11 realizations expanded symmetrically around its expectations have been performed. Gaussian probability distributions are adopted for all parameters, because for the given expectation and standard deviation, it causes the largest entropy (disorder) in the parameters subjected to randomization. This fact follows directly the Maximum Entropy Principle so that, in this particular case, we consider the highest possible uncertainty and its impact on the given system. Recovered structural responses in the form of nodal displacements and internal forces at some bottom leg elements have been recovered each time, resulting in 1,108,800 discrete data points for both towers to be processed further. Table 3 contains all the introduced input uncertain parameters with their expectations and their representations of these expectations. It is remarkable that Young modulus of the given aluminum alloy is three times smaller than that of the applied steel; all the deadloads have been set automatically using material densities. The mean values of the given parameters have been underlined. 

Structural random responses of skeletal towers have been carried out with the iterative generalized stochastic perturbation technique. The structural response functions (SRFs) previously recovered by the ABAQUS 2024 system have been used as the data source for this part of the work. This probabilistic approach and also two concurrent ones, whose description is to follow, have been implemented into the system MAPLE 2015. The input coefficient of variation of all random parameters has been fixed as 0.10, and the 10th-order perturbation technique has been implemented into the algebra system. Structural response functions have been approximated by up to the 10th polynomials using the WLSM as univariate functions of the input random parameter; for example, the elastic modulus for which they would have been proposed in a form shown in Equations (6) and (7). The final order of polynomial approximation has been adjusted to prevent overfitting and underfitting phenomena at the upper and lower bounds of the random parameter realization scheme shown in Table 3, and also to maintain a relatively low mean square error. Polynomial order of approximation has been then chosen uniformly for all discrete time steps of investigated structural responses, aiming for automatization of the procedure for further implementation into real-life monitoring of the structures.

## 4. Numerical Results

**Figure 4:**
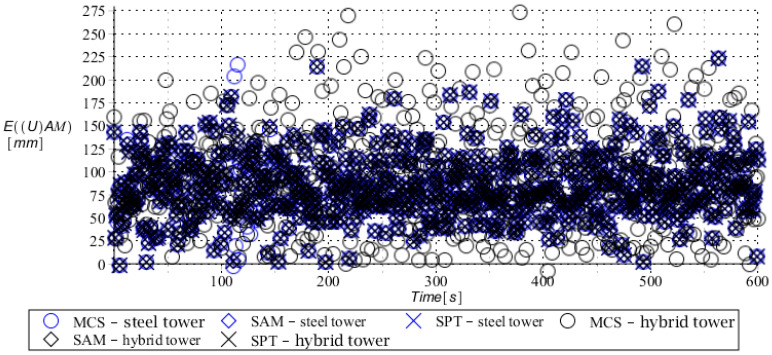
Expected value of horizontal displacement for steel tower (blue) and hybrid tower (black) with uncertain antenna mass.

**Figure 5:**
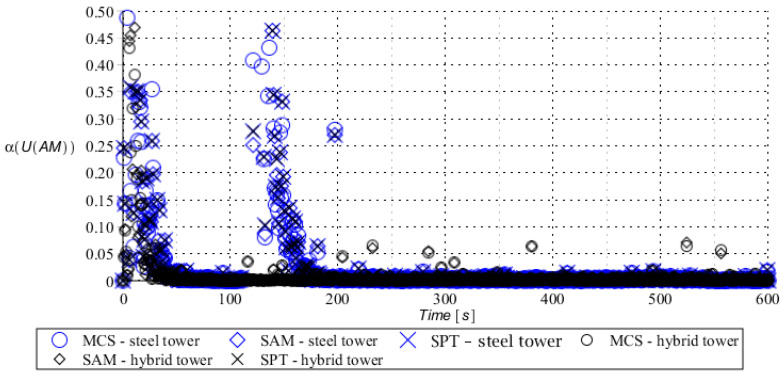
Coefficient of variation of horizontal displacement of steel tower (blue) and hybrid tower (black) with uncertain antenna mass.

**Figure 6:**
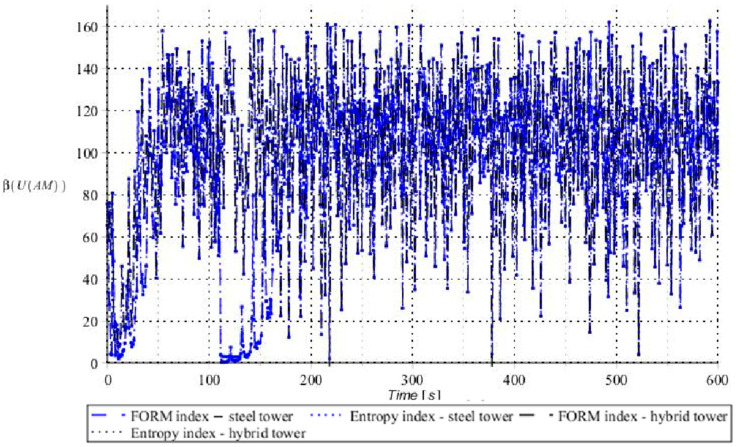
Reliability indices for SLS state of steel tower (blue) and hybrid tower (black) considering antenna mass as a random variable.

**Figure 7:**
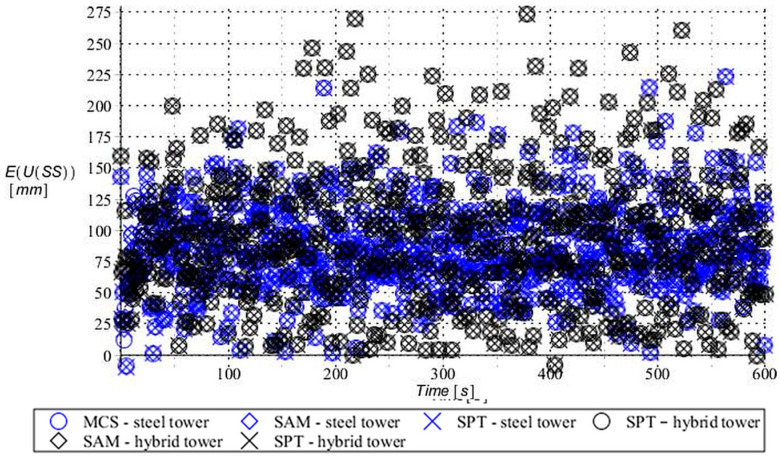
Expected value of horizontal displacements of steel tower (blue) and hybrid tower (black) with uncertain spring support stiffness.

**Figure 8:**
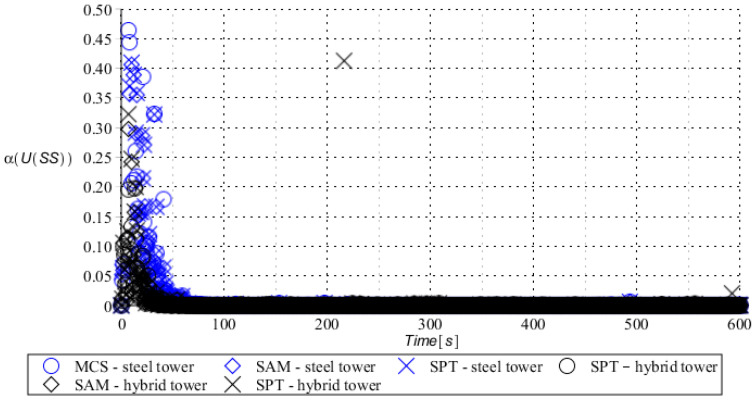
Coefficient of variation of horizontal displacements of steel tower (blue) and hybrid tower (black) with uncertain spring support stiffness.

**Figure 9:**
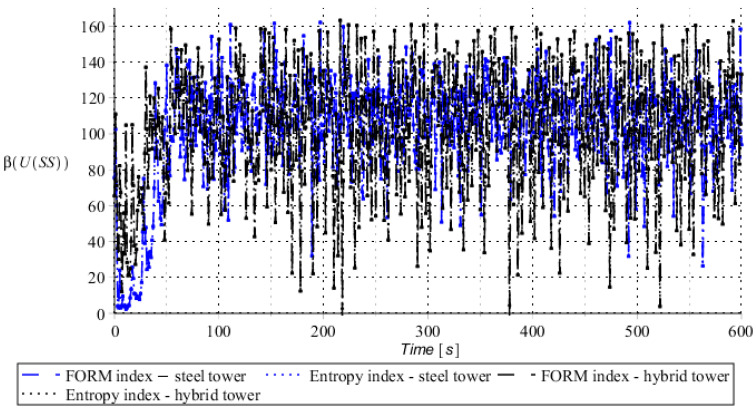
Reliability indices for SLS state of steel tower (blue) and hybrid tower (black) considering spring support stiffness as a random variable.

**Figure 10:**
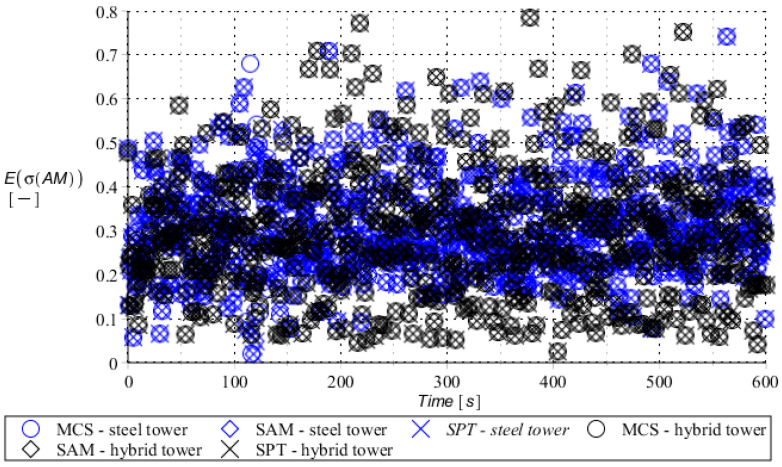
The expected value of the bearing capacity state of the compressed main leg of the steel tower (blue) and hybrid tower (black) with random antenna mass.

**Figure 11:**
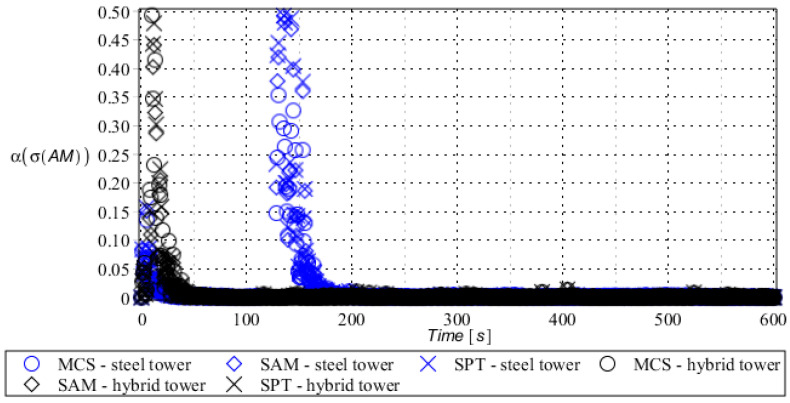
Coefficient of variation of bearing capacity state of the compressed main leg of steel tower (blue) and hybrid tower (black) with random antenna mass.

**Figure 12:**
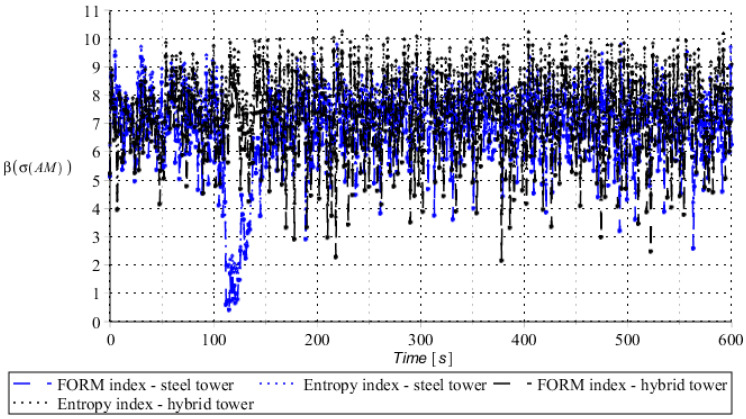
Reliability indices for ULS state of steel tower (blue) and hybrid tower (black) considering antenna mass as a random variable.

**Figure 13:**
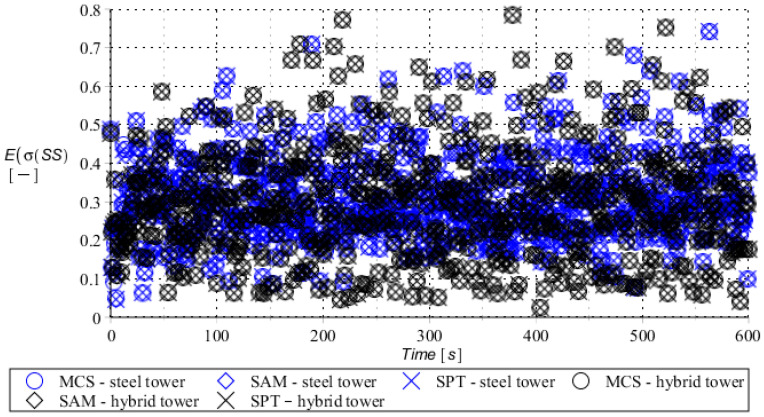
The expected value of the bearing capacity state of the compressed main leg of the steel tower (blue) and hybrid tower (black) with random spring support stiffness.

**Figure 14:**
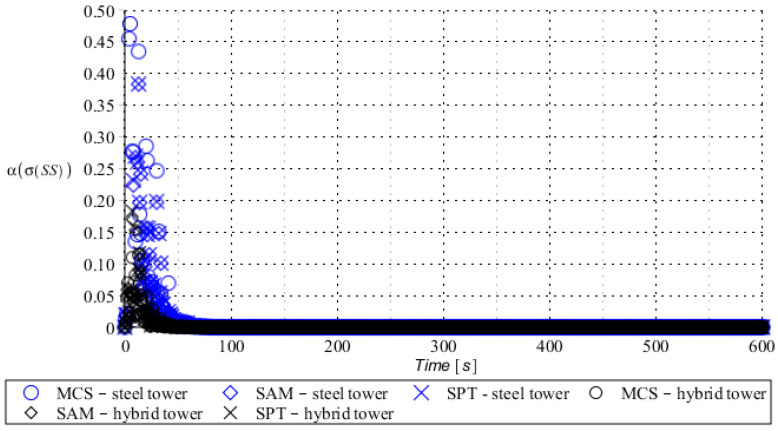
Coefficient of variation of bearing capacity state of the compressed main leg of steel tower (blue) and hybrid tower (black) with random spring support stiffness.

**Figure 15:**
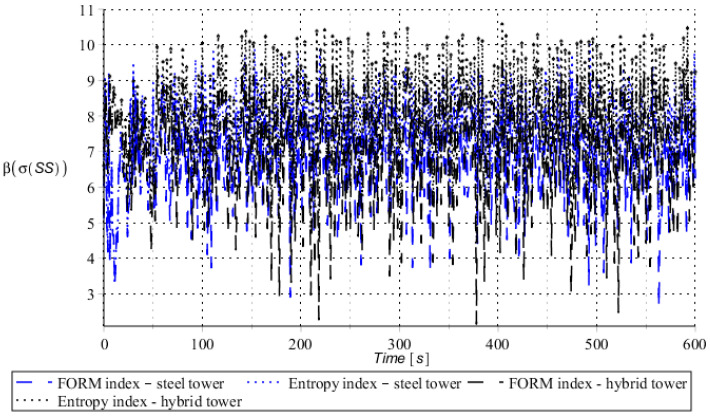
Reliability indices for ULS state of steel tower (blue) and hybrid tower (black) considering spring support stiffness as a random variable.

Numerical results presented in this work concern both the Ultimate Limit States (ULS) and the Serviceability Limit States (SLS) of investigated skeletal towers. The bearing capacity of the main leg of both towers has been considered in the ULS in the case, where the wind pressure acts symmetrically on two legs and one leg would undertake full compression of such an action; it is the most unfavorable state for the design of this member. The admissible horizontal displacement of the skeletal towers’ tops with the same wind pressure direction has been investigated in the ULS analysis. The admissible horizontal displacement has been assessed from the interrelation of the overall height of skeletal tower and the technical specification of its telecommunication equipment *H*/150. Numerical results have been contained in Figure 4, Figure 5, Figure 6, Figure 7, Figure 8, Figure 9, Figure 10, Figure 11, Figure 12, Figure 13, Figure 14 and Figure 15, where consecutively expected values, coefficients of variation, and reliability indices have been marked on the vertical axes in these graphs. The selected results include extreme probabilistic responses obtained while randomizing antenna masses (AM), as well as subsoil stiffness (SS). Horizontal axes of these graphs correspond to the given time interval, whereas the data series abbreviated by MCS, SAM, and SPT are equivalent to the Monte-Carlo, semi-analytical, and stochastic perturbation method, respectively. The data determined for the steel original tower are marked here with the blue series, while these adjacent to the hybrid tower—with the black symbols; “Cornell reliability index” series follow the FORM approach from Eurocode 0, while “New reliability index” correspond to the Bhattacharyya-based relative entropy rescaled to the same variability interval proposed in Equations (15) and (16).

First, it is seen that the expected values of structural responses approximated by the SPT show excellent accuracy in contrast to referential approaches for both studied limit states. Coefficients of variations (COVs) for the studied random structural responses exhibit similar accuracy patterns.

This allows us to pursue the aim of accurate structural reliability assessment for some dynamic wind fluctuations, which, in this case, have been assumed as changing with frequency of 1.0 Hz. Interestingly, the output random dispersion of structural responses has been generally smaller than the input one (smaller than 0.10). Some local peaks of output COV may be noticed during the entire analysis, which generally can be related to some local accuracy loss of the time integration scheme—this especially occurs at the propagation of simulations from the static case into a dynamic case, where the vector of nodal displacements is known, but the vectors of nodal velocities are gradually growing with some limited increase of nodal accelerations. This is handled by the algorithm in a manner that first initial time steps are taken by few orders of magnitude smaller than the admissible one. This significant decrease of time step interval of the method finally reflects on the obtained structural outputs. Moreover, a peak of output COV has been noticed in steel towers (Figure 11), which is also correlated with some inconsistency of output structural response dataset, which can be handled by shorter time steps, which, in turn, remarkably increases the overall computational cost. The reliability indices of the two analyzed types of towers are comparable for both SLS and ULS states under investigation for all different input random design parameters. Naturally, reliability indices for SLS of hybrid towers generally tend to differ more significantly when contrasted to the steel tower since the smaller elastic moduli of aluminum contribute to greater displacements of the top of the analyzed structure. The lighter upper segment contributed positively to the ULS state under consideration since the reduction of stresses in investigated sections of the lower steel segment of the hybrid tower has been observed. Some noticeable differences can be observed when two proposed formulations of the reliability index are contrasted, but their relative error only equals a few percent. The general trend that has been outlined by this study indicates that the reliability indices obtained using the relative probabilistic entropy concept are generally larger than those obtained by the Cornell idea, so that the FORM approach is a more restrictive safety measure.

With respect to the presented analysis, it can also be commented that some aspects of the design have not been investigated related to the significant differences between material properties of steel and aluminum. Thermal expansion coefficient differs in these materials, which should be carefully considered during the design of joints connecting the upper (aluminum) segment with the lower (steel) segment. Such differentiation in the thermal expansion coefficients may inflict additional shear in the bolted end plates of main legs connecting these two segments, which should be addressed as an additional load case (thermal load case) and included in the overall shear acting on these bolts to appropriately design their quantity and dimensions, as well as general joint geometry. That is why some temperature variations have been considered in numerical analysis and should be continued in case of heating of the entire towers.

## 5. Concluding Remarks

1. Probabilistic outputs in the form of expected values and coefficients of variation, as well as reliability indices, exhibit remarkable agreement between the results obtained independently with the iterative stochastic perturbation technique proposed in this study and Monte-Carlo simulations, and also the semi-analytical method that served as referential methods. At the same time, the stochastic perturbation technique exhibits remarkable computational efficiency when contrasted to other probabilistic approaches presented here. When contrasted with the semi-analytical approach, the probabilistic characteristics of the response with the reliability index estimation are obtained four times faster for the stochastic perturbation technique, whereas when contrasted to the Monte-Carlo simulations with 10^5^ random, trials this difference is greater than 30 times.
2. The very important aspect of this analysis is that the FORM reliability indices for the steel and hybrid steel–aluminum towers demonstrate generally very good coincidence. This is because the upper, softer part of the tower is not decisive for both limit states when rational optimization is delivered generally for these types of structures. This is because the upper, softer part of the tower is not decisive for both limit states when rational optimization is delivered. It generally confirms correctness of such an optimization direction. This also confirms its capability of replacing in further designing procedures of application of traditional steel skeletal structures with hybrid solutions. Further, one notices that the relative entropy based reliability indices in the ULS and SLS are very similar to their FORM-based counterparts. It confirms an applicability of such a non-conventional reliability measure in conjunction with the existing designing procedures. It is seen that the proposed approach of common application of the Stochastic Finite Element Method and relative entropy apparatus may be efficient in reliability assessment of other tall structures like masts, chimneys, and possibly high-rise buildings, but other limit states like fatigue-based ones should also be considered. However, in this case, a relative entropy-based reliability assessment needs to be checked for their connections, which may be the weakest link in some specific case studies.
3. The design of a hybrid aluminum–steel tower allowed almost 11% mass reduction, concerning the steel origin having the same height and other geometrical parameters, and these savings have been obtained without any loss of the tower structure’s reliability level. The upper segment of the tower, designed from aluminum, exhibits a significant reduction of the self weight of the structure and exhibits natural resistance to the corrosion process. On the other hand, aluminum exhibits significantly smaller elastic moduli, which reflects greater displacements of the structure, which has to be carefully considered from the serviceability perspective. Overall economic applicability of such a design approach should also involve the discrepancy between the cost of steel and aluminum, not only as raw materials but also involving welding processes, anticorrosive coating, and maintenance for the steel elements, which may vary significantly in between different regions. It would also be very interesting to incorporate into the given stochastic model of the hybrid tower both spatial and time cross-correlations of the wind and dynamic responses. Spatial cross-correlations may affect the final value of the reliability index calculated above, whereas time cross-correlations are applicable when time-dependent reliability is assessed.

## Figures and Tables

**Table 1:** Cross-sections and materials of the steel skeletal tower.

Segment No.	Segment Height	Legs Section	Legs Material	Bracing Section	Bracing Material
1	3.00 m	CHS 139.7 × 6.3	S355	L 120 × 80 × 8	S355
2	3.00 m	CHS 139.7 × 6.3	S355	L100 × 75 × 8	S355
3	3.00 m	CHS 127 × 6.3	S355	L100 × 75 × 8	S355
4	3.00 m	CHS 127 × 6.3	S355	L100 × 75 × 8	S355
5	3.00 m	CHS 127 × 5.6	S355	L90 × 60 × 8	S355
6	3.00 m	CHS 127 × 5.6	S355	L90 × 60 × 8	S355
7	3.00 m	CHS 108 × 5	S355	L60 × 60 × 5	S355
8	3.00 m	CHS 108 × 5	S355	L60 × 60 × 5	S355
9	2.50 m	CHS 101.6 × 3.2	S355	L60 × 60 × 5	S355
10	2.50 m	CHS 101.6 × 3.2	S355	L60 × 60 × 5	S355
11	2.50 m	CHS 88.9 × 3.2	S355	L60 × 60 × 5	S355
12	2.50 m	CHS 88.9 × 3.2	S355	L60 × 60 × 5	S355
13	1.20 m	CHS 76.1 × 3.2	S355	L60 × 60 × 5	S355
14	1.20 m	CHS 76.1 × 3.2	S355	L60 × 60 × 5	S355
15	1.20 m	CHS 76.1 × 3.2	S355	L60 × 60 × 5	S355
16	1.20 m	CHS 76.1 × 3.2	S355	L60 × 60 × 5	S355
17	1.20 m	CHS 76.1 × 3.2	S355	L60 × 60 × 5	S355
Total weight of the numerical model				5796 kg	

**Table 2:** Cross-sections and materials of the hybrid skeletal tower.

Segment No.	Segment Height	Legs Section	Legs Material	Bracing Section	Bracing Material
1	3.00 m	CHS 139.7 × 6.3	S355	L 120 × 80 × 8	S355
2	3.00 m	CHS 139.7 × 6.3	S355	L100 × 75 × 8	S355
3	3.00 m	CHS 127 × 6.3	S355	L100 × 75 × 8	S355
4	3.00 m	CHS 127 × 6.3	S355	L100 × 75 × 8	S355
5	3.00 m	CHS 127 × 5.6	S355	L90 × 60 × 8	S355
6	3.00 m	CHS 127 × 5.6	S355	L90 × 60 × 8	S355
7	3.00 m	CHS 108 × 5	S355	L60 × 60 × 5	S355
8	3.00 m	CHS 108 × 5	S355	L60 × 60 × 5	S355
9	2.50 m	CHS 110 × 6	AW-6061 T6	L80 × 80 × 6	AW-6061 T6
10	2.50 m	CHS 110 × 6	AW-6061 T6	L80 × 80 × 6	AW-6061 T6
11	2.50 m	CHS 110 × 4	AW-6061 T6	L80 × 80 × 6	AW-6061 T6
12	2.50 m	CHS 110 × 4	AW-6061 T6	L80 × 80 × 6	AW-6061 T6
13	1.20 m	CHS 90 × 5	AW-6061 T6	L80 × 80 × 6	AW-6061 T6
14	1.20 m	CHS 90 × 5	AW-6061 T6	L80 × 80 × 6	AW-6061 T6
15	1.20 m	CHS 90 × 5	AW-6061 T6	L80 × 80 × 6	AW-6061 T6
16	1.20 m	CHS 90 × 5	AW-6061 T6	L80 × 80 × 6	AW-6061 T6
17	1.20 m	CHS 90 × 5	AW-6061 T6	L80 × 80 × 6	AW-6061 T6
Weight of steel part (height 24.0 m) Weight of aluminum part (height 16.0 m)				4542 kg 622 kg	
Total weight of the numerical model				5164 kg	

**Table 3:** Uniform discretization of the uncertain parameters for the needs of the WLSM tests.

No.	Antenna Mass (Kilograms)		Elastic Moduli (GPa)		Spring Support Stiffness (N·m^−1^) × 10^7^		Profile Thickness	Temp.	Wind Velocity
	AS-5-20	VHLP2-18/B	S355	AW-6061 T6	Compressed	Uplifted	(−)	(°C)	(−)
1	4.50	9.680	189.0	63.00	5.200	1.895	0.900	−45	0.900
2	4.60	9.900	193.2	64.40	5.312	1.937	0.920	−46	0.920
3	4.70	10.12	197.4	65.80	5.428	1.979	0.940	−47	0.940
4	4.80	10.34	201.6	67.20	5.543	2.021	0.960	−48	0.960
5	4.90	10.78	205.8	68.60	5.659	2.063	0.980	−49	0.980
6	5.00	11.00	210.0	70.00	5.774	2.105	1.00	−50	1.00
7	5.10	11.22	214.2	71.40	5.889	2.147	1.02	−51	1.02
8	5.20	11.44	218.4	72.80	6.005	2.189	1.04	−52	1.04
9	5.30	11.66	222.6	74.20	6.120	2.231	1.06	−53	1.06
10	5.40	11.88	226.8	75.60	6.236	2.273	1.08	−54	1.08
11	5.50	12.10	231.0	77.00	6.351	2.316	1.10	−55	1.10

(−) uniform multiplier of the expected value.

## Data Availability

The research data can be obtained from the Authors upon the request.
